# Emergent Central Pattern Generator Behavior in Gap-Junction-Coupled Hodgkin-Huxley Style Neuron Model

**DOI:** 10.1155/2012/173910

**Published:** 2012-12-06

**Authors:** Kyle G. Horn, Heraldo Memelli, Irene C. Solomon

**Affiliations:** ^1^Program in Neuroscience, Stony Brook Universty, SUNY, Stony Brook, NY 11794-5230, USA; ^2^Department of Physiology and Biophysics, Stony Brook Universty, SUNY, Stony Brook, NY 11794-8661, USA; ^3^Department of Computer Science, Stony Brook Universty, SUNY, Stony Brook, NY 11794-4440, USA

## Abstract

Most models of central pattern generators (CPGs) involve two distinct nuclei mutually inhibiting one another via synapses. Here, we present a single-nucleus model of biologically realistic Hodgkin-Huxley neurons with random gap junction coupling. Despite no explicit division of neurons into two groups, we observe a spontaneous division of neurons into two distinct firing groups. In addition, we also demonstrate this phenomenon in a simplified version of the model, highlighting the importance of afterhyperpolarization currents (*I*_AHP_) to CPGs utilizing gap junction coupling. The properties of these CPGs also appear sensitive to gap junction conductance, probability of gap junction coupling between cells, topology of gap junction coupling, and, to a lesser extent, input current into our simulated nucleus.

## 1. Introduction

Central pattern generators (CPGs) correspond to neural regions that spontaneously generate oscillatory behavior in the absence of patterned input. In both invertebrates and vertebrates, they appear to play a critical role in the formation of repeated oscillatory behaviors, including activities such as walking, swimming, heartbeating, and breathing [1–4]. Because of their roles in cardiac and respiratory function, CPGs may be considered vital for basic survival across much of the animal kingdom.

Originally, the oscillatory behaviors seen in locomotion were presumed to be generated through reflexes alone. An ever-growing body of evidence, however, suggests that both locomotor [2, 5] and respiratory oscillatory activities [6, 7] are generated centrally in spinal cord and brainstem regions, respectively, since these behaviors occur in the absence of descending cortical drive and sensory input. Modulation of CPG activity, however, is necessary for adapting locomotor and breathing patterns to ever-changing environmental conditions. Because of this, both the locomotor [8] and the respiratory [9, 10] systems exhibit a great deal of plasticity in the face of changing conditions and, therefore, should be viewed as dynamic rhythm generating devices.

Traditionally, reciprocal synaptic inhibition between two neuronal populations (or two groups of neuronal populations, or even two individual neurons [11]) is seen as the standard method of generating CPG behavior in both biological and computational systems. Originally proposed by Brown [12], this style of CPG appears in biological models of lamprey [13] and stick insect locomotion [14]. It also appears in simulated salamander [15] and mammalian locomotion models [16] as well as in leech heart [17] and as a component in more complex models of respiratory activity [18]. This form of CPG is often referred to as the half-center model and is a prominent model for robotic locomotion controllers [19, 20].

While half-center CPGs typically focus on synaptic inhibition, recent work indicates that gap junction coupling may also play a role in locomotor patterns [21] and respiratory patterns in both amphibians [22] and mammals [23–26]. Gap junctions are a prominent mechanism for neuronal synchrony in the brainstem, cerebellum, and neocortex as well as among motoneurons, glia, and retinal cells [27–29], though they can also produce complex asynchronous patterns [30]. Gap junction proteins and functional gap junction coupling have also been demonstrated in numerous neurons associated with central respiratory control, including hypoglossal and phrenic motoneurons [25, 31] and the pre-Bötzinger complex [23, 32]. A combination of gap junctions and synaptic inhibition may also be responsible for synchrony in some neuronal populations [33], and even if gap junctions are not responsible for generating a mutually inhibitory connection, inhibitory currents coupled by gap junctions could easily play such a role.

Although hard-wired reciprocal synaptic inhibition may be easy to identify physiologically, we propose that this same style of inhibition can spontaneously form in a single pool of gap-junction-coupled neurons, mutually inhibiting one another via their slow afterhyperpolarization (sAHP). The slow sAHP following the action potential can be modified directly through calcium-gated potassium channels (called either *I*_AHP_ or *I*_SK_), which are found in many neurons including motoneurons [34, 35] and are known to play an important role in burst frequency modulation. Since neurons would dynamically assign themselves to one of the two “half-centers,” changes to gap junctions, *I*_AHP_, or inputs alone could modify how individual neurons align their firing. This would produce a highly dynamic modifiable half-center CPG capable of adapting to the rapid demands of locomotion or respiration.

Here, we present two biologically realistic models of gap-junction-coupled neurons that exhibit multiple output rhythms typical of half-center CPGs. Unlike standard half-center CPG models, however, we have one pool of ubiquitous neurons with random gap junction coupling that are still able to output two or more distinct phase-shifted rhythms.

## 2. Models

The full model is the hypoglossal motoneuron model developed by Purvis and Butera [36] and modified with gap junction coupling from Perez Velazquez and Carlen [37]. Despite the model's seeming specificity, many of the ion channels contained in this model also feature prominently in a variety of other neurons and motoneurons [35].

The reduced model is a combination of the simple spiking Izhikevich [38] *I*_Na_ + *I*_K_ model, which contains only a sodium, potassium, and leak current, with *I*_Ca_, [Ca^2+^]_*i*_, *I*_SK_, and gap junction coupling. The model itself for neuron *i*, where *i* = [1,2,…, *N*], is as follows:

(1)  CdVidt=Iinput,i(t)−g−leak(Vi−Eleak)      −g−Naminf⁡(Vi)(Vi−ENa)−g−Kni(Vi−EK)      −g−Capi(Vi−ECa)−g−AHPzi(Vi−EK)      −∑j=1Ng−gap⁡,i,j(Vi−Vj),minf⁡(V)=11+e(−26.5−V)/(14.5/ln⁡(5/3))  ,dnidt=ninf⁡(Vi)−niτn(Vi),ninf⁡(V)=11+e(−20−V)/5,  τn(V)=  3,dpidt=pinf⁡(Vi)−piτp(Vi),pinf⁡(V)=11+e(−40−V)/5,  τp(V)=61+e(55+V)/2+0.5,dzidt=zinf⁡(Vi)−ziτz(Vi),zinf⁡([Ca2+]i)=11+(0.003/[Ca2+]i)5,  τz(V)=10,d[Ca2+]i,idt=−0.0005  ICa−0.04  [Ca2+]i,i,ICa=g−Capi(Vi−ECa).

With the following parameters:

(2) $$\begin{array}{c} {\overset{-}{g}}_{\text{leak}}=0.38\;\mu \text{S},\;\;{\overset{-}{g}}_{\text{Na}}=1.28\overset{-}{3}\;\mu \text{S},\;\;\;{\overset{-}{g}}_{K}=1.8\;\mu \text{S},\\ {\overset{-}{g}}_{\text{Ca}}=0.08\;\mu \text{S},\;\;{\overset{-}{g}}_{\text{AHP}}=0.5\;\mu \text{S},\\ {\overset{-}{g}}_{\mathrm{gap},i,j}=\{\begin{array}{cc} g_{\mathrm{gap}} & \text{if}\;\text{neurons}\;i\;\;\text{and}\;j\;\;\text{share}\;\text{a}\;\mathrm{gap}\;\text{junction},\\ 0\;\mu \text{S} & \text{if}\;\text{neurons}\;i\;\;\text{and}\;j\;\;\text{are}\;\text{not}\;\text{connected}, \end{array}\\ E_{\text{leak}}=-80\;\text{mV},\;\;E_{\text{Na}}=60\;\text{mV},\\ E_{\text{K}}=-80\;\text{mV},\;\;E_{\text{Ca}}=80\;\text{mV},\\ N=100,\;\;dt=0.02\;\text{ms},\;\;C=0.04\;\text{nF}. \end{array}$$

Changes to default parameters in specific simulations will be given in the description of each simulation run.

## 3. Simulations and Analysis

Simulations were performed using the Python programming language (http://www.python.org/) accompanied by the Scientific Tools for Python package (http://www.scipy.org/). Speed-critical code was written in C++ and plugged into Python using the C-Extensions for Python library (http://www.cython.org/). Plotting was done via the matplotlib library (http://www.matplotlib.sourceforge.net). 

Numerical integration of both the full and reduced motoneuron models was done via Euler's method. Both models were also tested over a variety of dt settings, and against RK4, the most common Runge-Kutta method [39], to ensure sufficient numerical accuracy by Euler's method.

Simulated neurons were deemed part of a singular cohesive firing group based on clustering via the Expectation-Maximization algorithm [40], with the number of clusters determined by the following method: first, the voltage of all neurons was summed together at every point in time. Next, this summed signal was smoothed via six passes of a 4 ms square wave moving average filter. Finally, this signal was normalized, local maxima were determined by observing when the derivative passed through zero, and an extremely low threshold of 6% of the total signal was used to discard spurious local optima that sometimes appeared when only a few neurons were active. Because firing neurons have significant depolarizations both before and after firing, analysis of summed voltage traces proved superior in identifying firing groups in comparison to the popular information theoretic “jump” method [41]. In addition, no groups were deemed to exist if the average of all voltage traces failed to reach a maximum value of 3 mV (out of a possible ~21 mV), implying that fewer than 15% of all spikes were well aligned in the largest group.

For the full model, all simulations were all performed with a 500 ms duration square wave input current of 0.5 ± 0.35 nA, where the variation corresponds to white noise changing at every time step. Since increasing or decreasing white noise amplitude did not noticeably produce distinct changes in the model, other levels of white noise were not further considered. The current was applied to each neuron beginning at 100 ± 10 ms into the simulation and ending at 600 ± 5 ms. Variation in input current start and end times simulates the varied delays in stimulation produced by axonal variation of input drive. Gap junctions were opened at 300 ms for the demonstration of behavior in the full model, at 125 ms when reducing SK conductance in the full model, and at 200 ms when examining plane topology in the full model. Gap junctions were closed for all full models at 700 ms. Gap junction connections were made at random, with each cell having a probability of 25% of connecting to any other cell in the full model without plane topology, and 50% of connecting to any cell within a radius of 5 in the model with topology. Neighboring cells in the plane topology were all evenly spaced in a square lattice, with nondiagonal neighbors at a distance of 1. The precise gap junction connectivity generated to demonstrate the behavior in the full model was reused in the simulations with SK conductance in the full-model, so that results could be directly compared. In the full-model simulation with SK conduction reduction, maximal conductance began at 0.3 *μ*S. Starting at 300 ms, the conductance dropped in a linear value until hitting 0.0 *μ*S at 500 ms, where it remained at zero for the duration of the simulation. All full simulations lasted 700 ms, had a gap junction conductance setting of 0.0005 *μ*S, and consisted of 100 neurons.

For the reduced model, simulations were performed using a constant square wave impulse, beginning at 100 ± 200 ms, where negative start times imply a start time of zero and ending at 1400 ms. Input current for reduced SK conductance simulations was set to 0.08 nA and for plane topology simulations was set to 0.1 nA. Gap junction conductance *g*_gap⁡_ in plane topology simulations was set to 0.003 *μ*S. Gap junctions in all reduced simulations remained open for the complete duration of the simulation. Gap junction connectivity in all figures illustrating multiple runs with varying parameters was performed using the same set of gap junction connections to ensure that results are not a byproduct of different gap junction connectivity. The reduced model gap junction conductance versus input current experiments and the connectivity in the reduced model gap junction conductance versus input current simulations also used the same gap junction connections to ensure comparability between generated results. The total simulation time for all reduced model runs was 1500 ms.

In our theoretical treatment of gap junction connection probability and connectivity radius, the cutoffs for each firing group were chosen based on the final column of Figure 7(a). An average total gap junction current below 0.09 nA produced ungrouped firing, between 0.09 nA and 0.1 nA produced three groups, between 0.1 nA and 0.3 nA produced two groups, and above 0.3 nA produced one group. The constant *g*, the conductance of each gap junction, was set to 0.003 *μ*S, to match the conductance settings in the accompanying simulations. The voltage differential constant *dV*, which is arbitrarily defined, was set to $0.\overset{-}{3}\;\text{V}$  so that ∑_*j*=1_^*N*_*i*_^ *g* *dV* = 1/*N*_*T*_, where the total number of neurons in the simulation equals *N*_*T*_. This ensures the maximum for average total gap junction current under an infinitely large radius and all-to-all connectivity yields 1.0 nA of average total gap junction current.

## 4. Results

### 4.1. Half-Center-Like Behavior

**Figure 1:**
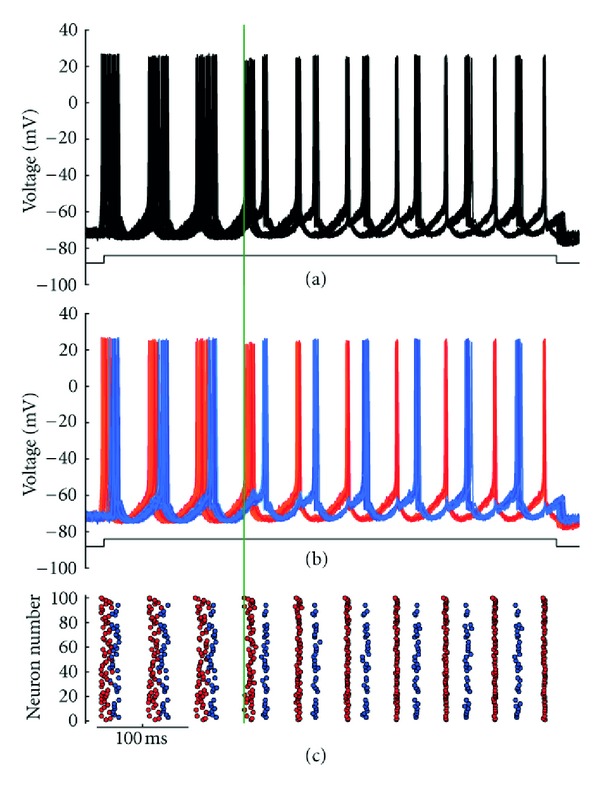
Example of half-center-like behavior in the full model. (a) Plot of voltage traces showing 40 of the 100 neurons. (b) Each voltage trace shown in (a) has been colored based on its neuron group affiliation. (c) A raster plot showing spikes for all 100 neurons, also colored based on neuron group affiliation. Vertical line corresponds to the opening of gap junctions.

In the full model, following the opening of gap junctions, two distinct phase-shifted signals reminiscent of a traditional half-center CPG could be generated in a single nucleus with ubiquitous connectivity. An example of this behavior is shown in Figure 1, where opening the gap junctions shifted a fairly asynchronous firing pattern amongst the 100 neurons into two distinct neuronal firing groups. To highlight this division into two firing groups, the neurons were color coded according to their group affiliation (Figure 1(b)), and a raster plot of their spiking behavior was generated (Figure 1(c)). In general, one group was often better aligned than the other, and upon further investigation, the randomness and ubiquity of connectivity actually fostered conditions encouraging one slightly larger group to act as a “driver” for the smaller “follower” group. On average, connectivity between and within the firing groups is given in Table 1, with the probability of two neurons being gap junction coupled set to *C*, and the number of neurons in firing group *i* being *S*_*i*_. 

While this implies that both groups are sending roughly an equal amount of conductance between one another, as would be expected by bidirectional gap junction coupling, it masks a more important property. Since each group has a very different degree of interconnectedness, the amount of incoming drive in relation to internal drive is markedly different (see Table 2).

As *S*_1_ ≫ 1 and *S*_2_ ≫ 1, the ratio of internal connections in group 1 is identical to the ratio of connections in group 2 received from group 1. Regardless the size of each spontaneously formed group, the larger group always receives more internal than external stimulation and extends more excitability to drive the smaller group. Thus, based on probability alone, a similar topology is consistently observed regardless of network size. This does not exclude the possibility of having two equally sized groups, which would be expected to have more balanced dynamics.

**Figure 2:**
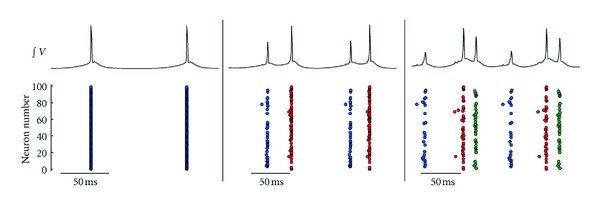
Examples of firing behaviors observed in the reduced model. One, two, and three firing groups through summation of all 100 neuronal voltage traces over time (upper panel) and raster plots of all spikes colored by group affiliation (lower panel).

While the main focus of the current investigation is on half-center like CPGs, it should be noted that generating more than two groups is entirely feasible. In the reduced model, for example, not only one or two centers could be generated, but also occasionally three centers were observed (Figure 2).

**Figure 3:**
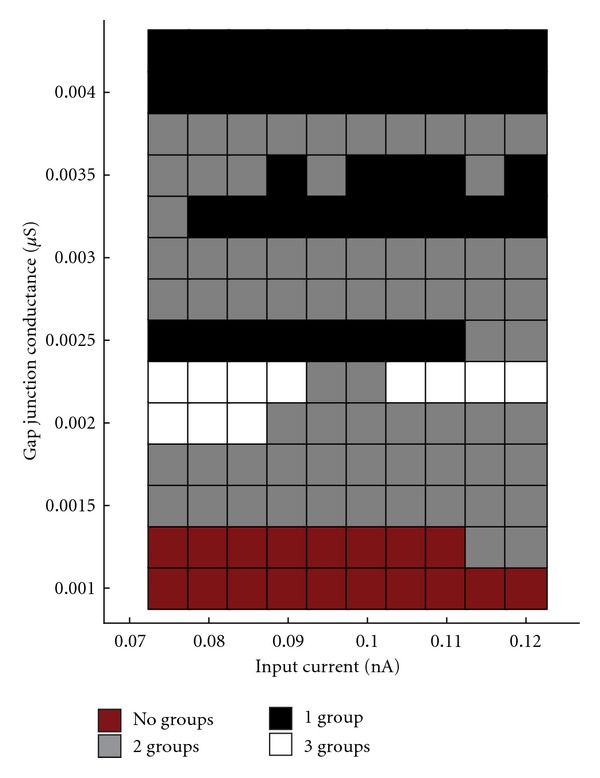
Gap junction conductance versus input current (reduced model). Multiple simulation runs examining the effects of changing gap junction conductance and input current on the formation firing groups.

Furthermore, even without altering patterns of connectivity, modifying conductance through gap junctions and/or input current into the system had the potential to shift the firing patterns between 1, 2, and 3 independent groups (Figure 3). In this case, gap junction conductance was seen to exert a greater influence than that of input current in determining the number of firing groups (Figure 3), with higher gap junction conductance being associated with fewer groups and lower gap junction conductance being associated with more groups. While input current was capable of producing a shift between different firing behaviors, changes in input current were less predictive of a trend in the number of firing groups.

### 4.2. Behavior Requirements

We hypothesized that *I*_AHP_ is a prime candidate for the necessary reciprocal inhibition between firing groups. Unlike other currents, *I*_AHP_ is solely gated by intracellular calcium. This delays the onset of the current until shortly after the spike ends and maintains the level of inhibition high until intracellular calcium is pumped out. Thus, the *I*_AHP_ is long lasting and easily modified by altering intracellular calcium influx. This would also serve to inhibit neighboring electrically coupled cells firing shortly after a spike but have less of an effect on neighboring cells firing concurrently. Without *I*_AHP_, the afterhyperpolarization and relative refractory period of the neuron are very brief. While it may be hypothetically plausible to use the relative refractory period generated by *I*_K_ or other comparatively brief negative currents for reciprocal inhibition, this is not observed in our models.

To explore the necessity and sufficiency of *I*_AHP_ on this behavior in our models, we first examine our reduced model. This model itself takes a simple spiking model (*I*_Na_ + *I*_K_) and extends it with the minimal requirements for a functional *I*_AHP_ channel: voltage-gated calcium currents (*I*_Ca_), intracellular calcium concentration ([Ca^2+^]_*i*_), and the AHP current (*I*_AHP_). The fact that this behavior is observed in the reduced model is a demonstration that, with the right parameters, the addition of a functional *I*_AHP_ current to a model is sufficient to produce more than one half-center like firing groups. However, since proper parameter setting is required, it is not sufficient alone to merely add a functional *I*_AHP_ current and expect half-center like CPG behavior.

**Figure 4:**
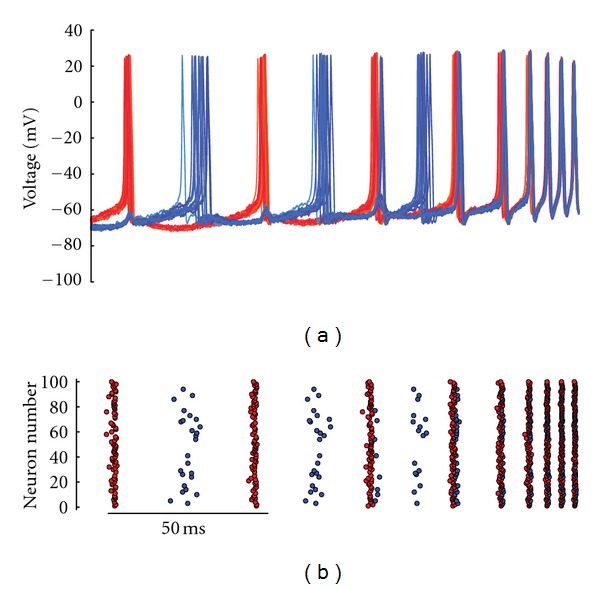
Effects of reduction and removal of *I*_AHP_ (full model). Simulation demonstrating that when ${\overset{-}{g}}_{\text{AHP}}$ is reduced linearly, the two firing groups merge into one group before ${\overset{-}{g}}_{\text{AHP}}$ reaches zero. This behavior can be observed in both (a) the voltage traces (shown for 40 of the 100 neurons) and (b) a raster plot of spikes for all 100 neurons. Both are color coded based on their firing group affiliation prior to the merging of the firing groups.

**Figure 5:**
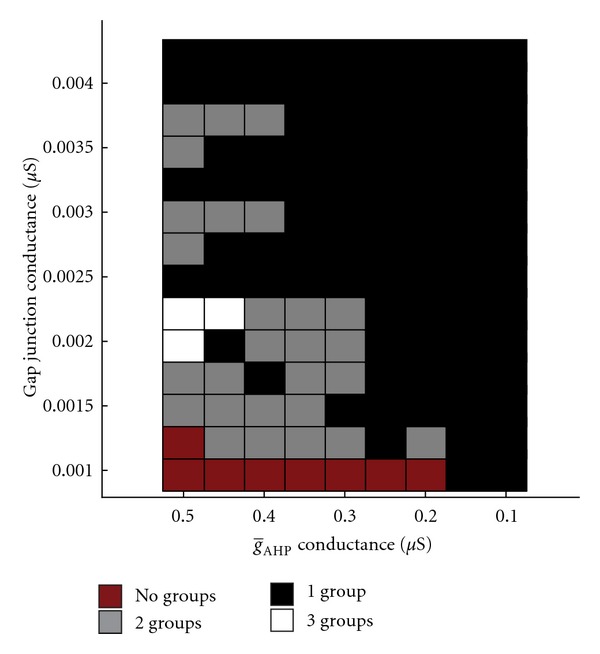
Gap junction conductance versus ${\overset{-}{g}}_{\text{AHP}}$ conductance (reduced model). When multiple simulations are performed with varying gap junction conductances and values for ${\overset{-}{g}}_{\text{AHP}}$, the incidence of having more than one firing group is abolished before reaching ${\overset{-}{g}}_{\text{AHP}}=0$. Note that the leftmost column of this figure corresponds to the input current = 0.08 nA column in Figure 3.

Based on our models, when *I*_AHP_ is gradually removed, the behavior ceases. In the full model, at sufficiently low *I*_AHP_, the two firing groups merge into one (Figure 4). Greater detail is shown based on simulations from the reduced model, where changes to *I*_AHP_ were compared with the primary determinant of group size: gap junction conductance (Figure 5). In both the full and reduced models, multiple firing groups could not be maintained in the absence of *I*_AHP_. Trivially, without the existence of gap junctions, neurons would be completely uncoupled and, thus, incapable of synchronizing.

### 4.3. Topology

**Figure 6:**
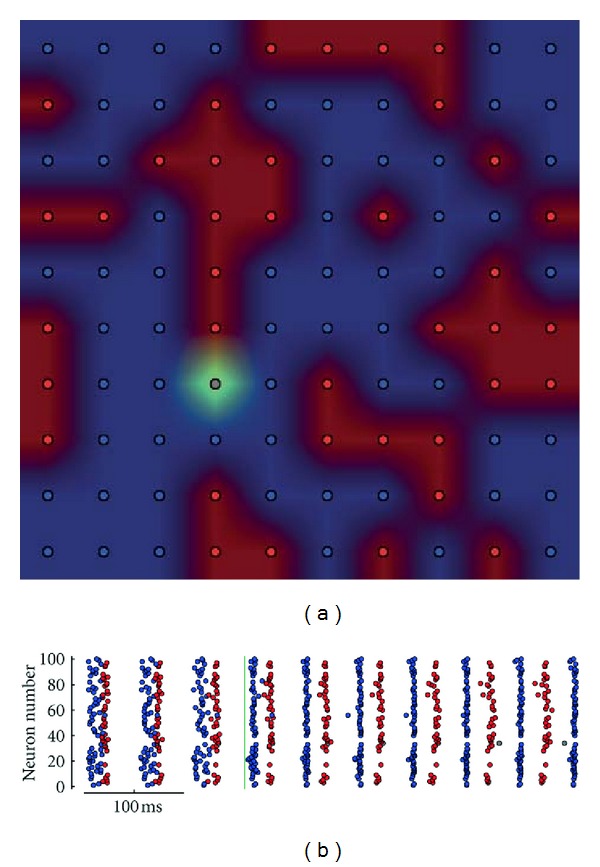
Mottled topology (full model). Demonstrations of a simulation run with a planar topology and a connectivity radius of 5. In (a), each neuron is plotted in its correct topological orientation and colored based on its group alignment. In (b), a raster plot confirms that these neurons are indeed split into two firing groups. The one grey/green neuron began as a member of the red firing group, but later transitioned to the blue group after several cycles of firing.

Up until now, all of our models have used random connectivity without any concern for spatial placement of the cells. Since slice preparations are commonly used to study CPGs of the spinal cord [42], and the slice itself often has a thickness (350–600 *μ*m) within the range of the dendritic span of motoneurons (250–700 *μ*m) [43], where gap junctions primarily form between dendrites and/or somas, we opted to orient neurons along a two-dimensional plane as a first approximation to this layout to begin to explore the effects of spatial connectivity on half-center like CPG group formation. While one might predict that neurons in each firing group would clump together into two massive nuclei, this is not the case. Instead, neuronal groups tended to form a mottled appearance, with clumps from each group equally interspersed (Figure 6). This configuration would ensure that each neuron would be exposed to some members of each firing group, thus loosely preserving connectivity reminiscent of a topology-free model. Figure 6 also reveals that one neuron spent five firing cycles with the firing group color coded in red before joining the firing group color coded in blue. This suggests that unlike in a traditional half-center CPG where group allegiance is a hard-set property of each neuron, in some rare instances, a neuron may straddle the fence and move between two groups.

**Figure 7:**
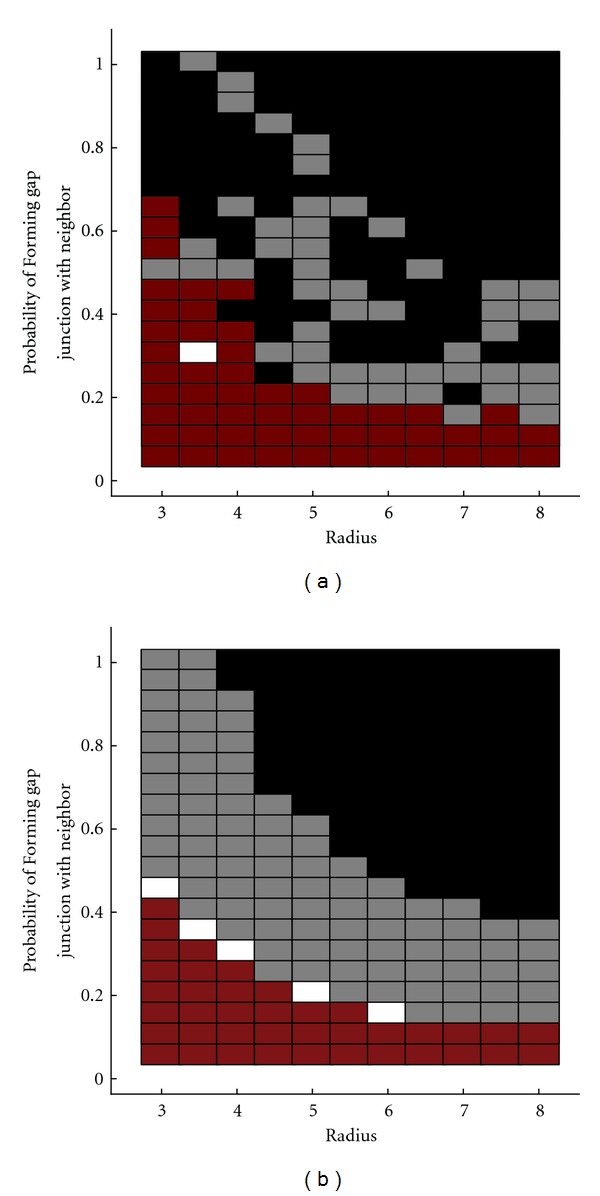
Connectivity topology versus radius (reduced and theory). (a) Multiple simulations are performed varying both the probability of forming a gap junction with a neighbor within a given radius and the radius itself. The results from this series of simulations resembles (b) a plot based on the average of the total hypothetical conductance received by each neuron.

Finally, using the reduced model, we explored the parameter space to further examine the exact role that topology plays (Figure 7(a)). We found that the pronounced inverse relationship between connectivity probability and connectivity radius is best explained by overall gap junction current. We reasoned that by imposing a radius of connectivity, the number of available neurons to form gap junctions would be decreased, and the net current entering all gap junctions for each individual cell would be decreased. If we assume that every pair of cells has an identical voltage differential *dV*, probability *C* of connecting to other neurons within a radius *R*, and a gap junction conductance *g*, and also assume a plane of neurons of infinite size and some total current “window” where two firing groups can exist, we can generate a theoretical prediction of when two firing groups will form (Figure 7(b)). If we represent the number of neurons within a radius *R* of neuron *i* to be *N*_*i*_ neurons, and the total number of neurons to be *N*_*T*_, then the equation for average total conductance is as follows:

(3) $$\begin{array}{c} \langle I_{\mathrm{gap},\text{total}}\rangle =\frac{\sum_{i=1}^{N_{T}}I_{\mathrm{gap},i,\text{total}}}{N_{T}}=\frac{\sum_{i=1}^{N_{T}}\left(C\sum_{j=1}^{N_{i}}gdV\right)}{N_{T}}. \end{array}$$

## 5. Discussion

In this study, we have elucidated the properties of a novel type of CPG that oftentimes bear resemblance to the traditional half-center CPG depicted in the literature. Unlike most traditional models of CPGs, we have presented a more dynamic entity that allocates group membership on the fly and can modify its firing properties through a variety of different biological parameters. This property itself has a number of benefits and drawbacks.

It cannot be stressed enough that the dynamic nature of these CPGs would be especially beneficial for either the generation or modification of locomotor or respiratory central patterns. In contrast to our model, most models of CPGs incorporate static group affiliation, which alone may not be able to produce the sorts of dynamically changing locomotor and respiratory patterns seen in nature. In both locomotion and respiration, adaptation of rhythms to both external environmental changes and descending cortical commands may be more difficult in a simpler CPG, which may lack the requisite complexity required to describe the wealth of patterns that humans and other mammals are capable of exhibiting in these two activities. Moreover, CPGs formed through gap junctions can alter group affiliation without relying explicitly on changes in gap junction coupling. With this in mind, some of the rarer behaviors seen in the current model, including the more exotic three firing group behavior, might be easy for a biological system to generate and maintain as long as the initial state of the system is within the vicinity of the correct set of parameters. Evolutionarily speaking, it would also be easier to create a CPG that itself had no explicit wiring, but could rely on random connectivity to self-organize.

Though this novel CPG has many admirable traits, some inherent properties of these systems may make them harder to tune or more difficult to find biologically. The volatile nature of a system that drastically changes behavior with small changes in parameters could open such neural systems up to a plethora of neurological disorders. While we offer no strong hypotheses regarding known disorders that might stem from such a disruption, known disorders with errant or absent patterns certainly come to mind: spastic gait, persistent muscle spasms, and the sudden loss of breathing implicated in SIDS. Furthermore, because such systems can exist in a singular nucleus with otherwise ubiquitous physiological properties, the only way to identify such systems experimentally would be to observe them while active, rather than through simple histology alone.

### 5.1. Fast Pattern Generation

At first, the speed of oscillations in our proposed CPG may appear surprisingly fast, commonly ranging between 10 and 30 Hz. However, these speeds are not uncommon for many smaller animals in locomotion, respiration, and associated behaviors. For example, depending on its speed, the American Cockroach (*Periplaneta americana*) commonly has a stride frequency between 20 and 25 Hz, which at the highest speeds is often quadrupedal or bipedal, and only a few Hz below wing beat frequency [44]. In addition, the wing beat frequency of the ruby-throated hummingbird (*Archilochus colubris*) nearly doubles this at 53 ± 3 Hz. [45]. At slightly lower frequencies, but still within this range, are whisking behaviors in rats (at 6–12 Hz) [46] and mice (at 19 ± 7 Hz) [47] as well as sniffing behaviors in rats (at ~8 Hz) [48] and mice (at ~12 Hz) [49], both of which are commonly associated with respiratory events. Finally, hypoglossal motoneurons, the motoneuron corresponding to our full model, have been observed with steady-state frequencies as low as 9.7 ± 3 Hz and as high as 70+ Hz [50]. While all the aforementioned frequencies are within one standard deviation of the lowest frequencies observed in hypoglossal motoneurons, slow motoneurons in the cockroach (*Blaberus discoidalis*) leg can fire even slower, at 2–5 Hz [51]. Given the broad range of behaviors that fall within the firing frequencies observed in both the model and biological neurons, we believe that our proposed CPG represents a reasonable approach for modeling a variety of locomotor and respiratory behaviors.

### 5.2. CPG or Downstream Modifier?

While we have focused on pattern generation, our proposed CPG could also just act as easily as a pattern modification nucleus. Rather than using multiple firing groups to drive two separate sets of muscles, for example, it could be used to take a singular signal and double or triple its period. Considering the ability to rapidly swap between one and two firing groups, this could be especially useful in locomotor transitions, such as moving from walking to running, or in cortical processes used to keep track of time.

### 5.3. Limitations

As is the case with all modeling, a reasonable computational model does not necessarily imply the existence of a biological correlate. In addition, we are limited to the sets of parameters explicitly examined, and, therefore, we cannot rule out that additional exotic effects may not exist for this style of CPG. While we have tried to keep biological plausibility in the forefront, the lack of plasticity in our models could, in theory, lead us to miss key properties of these systems.

### 5.4. Inhibitory Synapses

While the current models stress a role for *I*_AHP_ and gap junctions, similar outcomes in terms of emergent topology should be obtainable via synaptic connections. Recall that the most important property in our plane topology networks was total current flowing between cells. Thus, a benefit of inhibitory synapses would be that inhibition comes from neuronal firing rather than electrophysiological afterhyperpolarization. This would take the focus away from ion channels controlling the sAHP and instead focus on mechanisms of firing. Subsequently, this may still include channels associated with *I*_AHP_, as they are strongly associated with modifications to firing frequency [35], but other currents, such as the hyperpolarization-activated current (*I*_*h*_), may end up playing a more substantive role under inhibitory synapses, as observed by Marder and Bucher [2].

While synaptic connectivity may favor distally located cells in a CPG, gap junction coupling would certainly emphasize less broadly spaced neurons. This would make gap-junction-based CPGs more amenable to our CPG model, where connectivity is more dense and arbitrary, than spaced and planned out meticulously. The latter of which would likely be more favorable to an inhibitory synaptic design, as axons have reasonably high target specificity. Thus, synaptic-based versus gap-junction-based CPGs may favor different styles of design.

### 5.5. Emergent Connectivity

It is easy to get into the habit of viewing the brain as a large hard-wired microchip; however, such analogies neglect the lively self-organized properties of the brain that could lead to unique emergent behaviors, like those demonstrated by our proposed CPG. While highly deterministic behaviors can arise from arbitrary connectivity, “dynamic” should not necessarily be conflated with “chaotic.” Even in the most volatile systems, the laws of probability can impose order and, in some cases, may yield a more stable design paradigm than the one that is seemingly more orderly. Our simulations with random gap junction coupling clearly illustrate this by demonstrating that regardless of the size of each group, the connectivity always scales to ensure that one group acts as the leader and the other follows in suit. Such design principles are simple to implement, even if they are not obvious at first.

### 5.6. Connectivity versus Radius: Simulation and Theory

Our theoretical predictions reasonably capture the various regions in parameter space where zero, one, two, and three firing groups typically appear (Figures 7(a) and 7(b)). Certainly the predictions do not perfectly match with the simulations, but the dynamic nature of these models makes absolute knowledge of when a certain number of firing groups appear difficult to define. Moreover, because the cutoff conductances need to be specified in our theoretical predictions for when each set of firing groups would appear, it cannot be seen as a predictive theory so much as an explanatory one. If one knew roughly under what total conductances certain firing groups would form, then that alone would be sufficient to detail a great deal of how many firing groups would be likely to appear in other simulation runs with different parameters.

### 5.7. The Benefits of Full and Reduced Models

By using both full and reduced style models, we have been able to gain the benefits of both. With the full model, we have a demonstration of our style of CPG in a model that has, otherwise, been deemed accurate to the biology. Clearly, the plethora of channels in a realistic neuron does not disrupt the ability to form multiple neuronal groups. Simultaneously, with our reduced model, we have stripped down the neuron to just the bare essentials to demonstrate the minimum required to achieve the desired behavior. Additionally, we gain the speed benefits traditionally associated with reduced models, which permits sufficient simulation runs for the many parameter versus parameter plots generated as part of this study.

## 6. Conclusions

The formation and modulation of centrally generated signals for essential behaviors remains an exciting and open field. Simply knowing where CPGs might lie in the brain may not be enough to fully understand exactly how they create their signals. Likewise, there may exist useful methods for pattern generation not used in nature that may still be useful for robotics and artificial prostheses. Thus, it is of great importance to explore the theoretical possibilities, so that we can provide biological experimentalists and roboticists ample paradigms from which to choose. While we have demonstrated a novel type of CPG, relying less on deliberate connections and containing no synapses at all, there may still exist other unique methods for generating these signals that are not yet addressed by the CPG literature or the current study. Thus, it is important to keep an open mind about the possibilities of how a CPG might form.

## Figures and Tables

**Table 1:** Total connections.

	From group 1	From group 2
Connections to group 1	*C*(*S*_1_^2^ − *S*_1_)/2	*CS*_1_ *S*_2_/2
Connections to group 2	*CS*_1_ *S*_2_/2	*C*(*S*_2_^2^ − *S*_2_)/2

**Table 2:** Percent connections.

	From group 1	From group 2
% Connections to group 1	(*S*_1_ − 1)/(*S*_1_ + *S*_2_ − 1)	(*S*_2_)/(*S*_1_ + *S*_2_ − 1)
% Connections to group 2	(*S*_1_)/(*S*_1_ + *S*_2_ − 1)	(*S*_2_ − 1)/(*S*_1_ + *S*_2_ − 1)
